# Efficient Online Controller Tuning for Omnidirectional Mobile Robots Using a Multivariate-Multitarget Polynomial Prediction Model and Evolutionary Optimization

**DOI:** 10.3390/biomimetics10020114

**Published:** 2025-02-14

**Authors:** Alam Gabriel Rojas-López, Miguel Gabriel Villarreal-Cervantes, Alejandro Rodríguez-Molina, Jesús Aldo Paredes-Ballesteros

**Affiliations:** 1 Mechatronics Section, Postgraduate Department, OMD Laboratory, Instituto Politécnico Nacional—Centro de Innovación y Desarrollo Tecnológico en Cómputo, Mexico City 07700, Mexico; arojasl2101@alumno.ipn.mx (A.G.R.-L.); jparedesb190@alumno.ipn.mx (J.A.P.-B.); 2 Colegio de Ciencia y Tecnología, Universidad Autónoma de la Ciudad de México, Mexico City 06720, Mexico

**Keywords:** online controller tuning, omnidirectional mobile robot, polynomial response surface method, evolutionary optimization

## Abstract

The growing reliance on mobile robots has resulted in applications where users have limited or no control over operating conditions. These applications require advanced controllers to ensure the system’s performance by dynamically changing its parameters. Nowadays, online bioinspired controller tuning approaches are among the most successful and innovative tools for dealing with uncertainties and disturbances. Nevertheless, these bioinspired approaches present a main limitation in real-world applications due to the extensive computational resources required in their exhaustive search when evaluating the controller tuning of complex dynamics. This paper develops an online bioinspired controller tuning approach leveraging a surrogate modeling strategy for an omnidirectional mobile robot controller. The polynomial response surface method is incorporated as an identification stage to model the system and predict its behavior in the tuning stage of the indirect adaptive approach. The comparative analysis concerns state-of-the-art controller tuning approaches, such as online, offline robust, and offline non-robust approaches, based on bioinspired optimization. The results show that the proposal reduces its computational load by up to 62.85% while maintaining the controller performance regarding the online approach under adverse uncertainties and disturbances. The proposal also increases the controller performance by up to 93% compared to offline tuning approaches. Then, the proposal retains its competitiveness on mobile robot systems under adverse conditions, while other controller tuning approaches drop it. Furthermore, a posterior comparison against another surrogate tuning approach based on Gaussian process regression corroborates the proposal as the best online controller tuning approach by reducing the competitor’s computational load by up to 91.37% while increasing its performance by 63%. Hence, the proposed controller tuning approach decreases the execution time to be applied in the evolution of the control system without deteriorating the closed-loop performance. To the best of the authors’ knowledge, this is the first time that such a controller tuning strategy has been tested on an omnidirectional mobile robot.

## 1. Introduction

### 1.1. Background

The use of mobile robots has significantly increased in recent years in different application fields such as educational, entertainment, military, industrial, and security sectors, among others [1,2]. In this context, omnidirectional mobile robots are among the most useful systems in those sectors because they are flexible, i.e., they can move in any direction independently of their orientation; hence, the controller performance is a fundamental issue that needs to be resolved for these robots to explore and navigate in complex and uncertain environments with abruptly changing disturbances [3]. So, omnidirectional wheeled mobile robots are more susceptible to disturbances and uncertainties during operation; therefore, their control systems need to be robust, reliable, and useful. Linear, nonlinear, and intelligent control systems have been designed to govern mobile robot dynamics, such as a linear-quadratic regulator (LQR), sliding-mode control, a neural controller, and adaptive observers [4,5,6,7].

Nevertheless, one key factor to improve controller performance is the selection of the controller parameters, referred to as “controller tuning”. Among the controller tuning strategies, adaptive alternatives (according to the taxonomy described in [8,9]) are the most outstanding approaches to deal with parametric uncertainties and external disturbances. This approach computes the controller parameters online (during system execution) and is sometimes called online controller tuning. One adaptive controller tuning approach, the indirect adaptive controller tuning approach (IACTA), uses an identification–prediction process to search for the solution. The IACTA [10] requires the analytical model of the system to be controlled. Recently, bioinspired optimization has been leveraged to iteratively determine model parameters during the identification stage and tune the controller parameters during the prediction stage. This approach is particularly effective as these algorithms excel in handling highly complex problems. The dynamic model requirement may be the reason that nowadays, the IACTA is applied to simple dynamics [10,11] due to the high computational load required to iteratively update the controller parameters, making its application difficult for autonomous mobile robots, considering the restrictions of an on-board computer.

### 1.2. Surrogate Models and Optimization Algorithms

In the last decades, surrogate models have been leveraged in the computational research field to aid the high computational burden of different optimization algorithms [12]. In particular, population-based bioinspired algorithms present application improvements when utilized with surrogate models. This is because traditional population-based bioinspired algorithms consist of an exhaustive search where multiple evaluations of a model are carried out [13]. If this model is too complex, the computational burden of well-known population-based bioinspired algorithms becomes unmanageable. Therefore, recent works in diverse research fields have employed and developed surrogate model-based bioinspired algorithms as in [14,15,16,17,18,19]. These implementations make it possible to address highly complex problems where the model to evaluate is computationally expensive or even nonexistent.

Using surrogate models with optimization algorithms (not only bioinspired) has become a common trend in control tuning strategies in the last decade. The current taxonomy is offered to appreciate the trends of these implementations in control tuning approaches [20]:

- Surrogate optimization-based controller tuning approach (SOCTA): This group considers the controller tuning process as an optimization problem solved offline to set the controller parameters and to implement them into the system for task execution in the second stage. These strategies can propose different performance criteria in the optimization problem. Their main characteristic is using a surrogate model in the offline optimization problem. Moreover, if bioinspired optimization algorithms are employed, these strategies can deal with highly complex dynamic models. Once obtained, the surrogate model and controller parameters remain fixed throughout the system’s execution.
- Surrogate adaptive controller tuning approach: Unlike the SOCTA, this one computes the controller parameters online (during the system’s execution). Similar to the previous tuning approach, this method also utilizes a surrogate model; however, it is now applied in online optimization. Bioinspired optimization algorithms are recurrently used to find new controller parameters because these can efficiently handle uncertainties and disturbances in the environment. The surrogate adaptive controller tuning strategies can be divided into two variants:
  – Surrogate direct adaptive controller tuning approach (SDACTA): This variant considers an approximated reference model acquired before the system’s execution by setting a surrogate model. This approximated reference model is compared against the real system during its execution, and the computed difference is used to update the controller parameters. The surrogate model contains fixed values during the system’s execution while the controller parameters are updated iteratively. Since the surrogate model is obtained before the system’s execution, the SDACTA remains subject to the model.
  – Surrogate indirect adaptive controller tuning approach (SIACTA): This computes both the surrogate model and controller parameters iteratively in short intervals during the system’s execution, making it less susceptible to parametric uncertainties and external disturbances. The surrogate model and the controller parameters change iteratively during the system’s execution.

This work is interested in the surrogate indirect adaptive controller tuning approach. The literature on this approach is presented in the next section.

### 1.3. State-of-the-Art Review

Table 1 summarizes eighty-five works related to surrogate controller tuning classification in the last fifteen years. The first column presents the surrogate-based controller tuning approach, while the second column lists references identified through a comprehensive search. Pie graphs are portrayed in the third column, where the number of works per surrogate model is displayed (some works employ more than one surrogate model). Also, it is noteworthy that within the pie graphs, the surrogate models evaluated are the Gaussian process (GP), Radial Basis Function (RBF), response surface method (RSM), Support Vector Machine (SVM), Artificial Neural Networks (ANNs), k-Nearest Neighbors (kNNs), and Decision Tree (DT). The unemployed pattern recognition techniques per controller tuning approach are displayed in red in the same column. Finally, the fourth column presents histograms illustrating the growth of surrogate-based controller tuning approaches over recent years. The following remarks on the above information are provided:

- The collection of works shows that the SOCTA is the most implemented approach with 53.48% of the works (46 out of 86), followed by the SDACTA with 32.55% (28 out of 86) and lastly the SIACTA with 16.27% (14 out of 86).
- The histograms show a clear increment in recent years regarding the application of surrogate-based controller tuning approaches. This trend is more evident in the SOCTA and the SDACTA. Nonetheless, the SIACTA (the current work’s method of interest) remains relatively unexplored.
- Grouping the surrogate models, it can be seen that the GP is significantly the most employed with 53.48% of the works (46 out of 86). This is attributed to its connection with the Bayesian optimization algorithm, which requires fewer function evaluations than traditional metaheuristic algorithms. However, in [21], it is shown that fewer function evaluations do not imply a faster solution. Also, it is remarkable that the kNN surrogate model has not yet been implemented in any controller tuning approaches, even if it has been successfully utilized in other black-box optimization problems.
- Concerning the current work’s tuning method of interest, the SIACTA, it is observed that the most employed surrogate model is the GP with 46.15% of the works (6 out of 13), followed by the RBF with 23.07% (3 out of 13). Furthermore, it is noteworthy that just one study [22] uses a single-neuron ANN due to the significant computational load of updating parameters as networks grow, rendering them unfeasible for experimental application. On the other hand, in only three recent works, the RSM surrogate model is incorporated in the SIACTA for brushless direct current motors [20] and for two- and three-degrees-of-freedom planar robotic manipulators [23,24]. Further research is needed to determine whether the benefits of the SIACTA based on the RSM hold true for increasingly complex autonomous mobile robots and whether closed-loop performance remains successful even as the system’s complexity rises.

**Table 1:** Surrogate-based controller tuning approaches summary.

Approach	References	Surrogate Models	Histogram
SOCTA	[25,26,27,28,29,30,31,32,33,34,35,36,37,38,39,40,41,42,43,44,45,46,47,48,49,50,51,52,53,54,55,56,57,58,59,60,61,62,63,64,65,66,67,68,69,70]	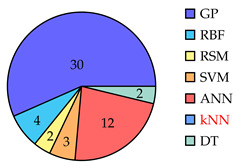	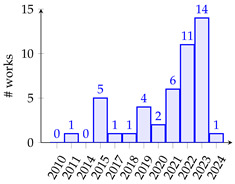
SDACTA	[71,72,73,74,75,76,77,78,79,80,81,82,83,84,85,86,87,88,89,90,91,92,93,94,95,96,97,98]	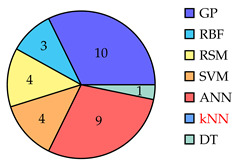	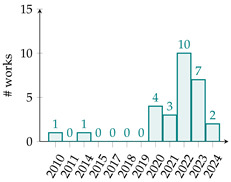
SIACTA	[20,22,23,24,99,100,101,102,103,104,105,106,107,108]	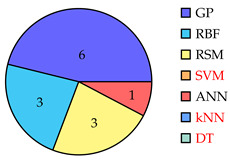	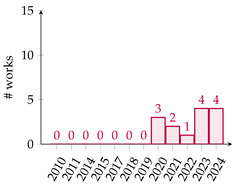

### 1.4. Contributions

Studies within the last decade have used surrogate tuning of the control system based on pattern recognition methods to lessen the computational burden when simulating dynamic systems. Few of these studies (16.27% of 86 works) have implemented the surrogate indirect adaptive controller tuning approach (SIACTA), where one of the less studied surrogate models is the response surface method (RSM). The SIACTA based on the RSM is a recent approach proposed by the authors of this paper [20] with only 3.48% of the works (three works). This approach has been appropriated in electromechanical systems [20] and in robotic manipulators [23]. The lack of studies related to the indirect adaptive controller tuning approach based on the surrogate model (SIACTA) with the response surface method in autonomous omnidirectional mobile robots makes it suitable to develop controller tuning solutions in this current gap in the state of the art. This study also provides a more efficient SIACTA strategy that better trades off the control performance and computation time, which is helpful for its incorporation into the embedded systems of autonomous mobile robots.

Then, the main contribution of this research is the development of the surrogate indirect adaptive controller tuning approach based on the response surface method (SIACTA-RSM) for omnidirectional mobile robots (OMRs). To emphasize the advantages of the SIACTA-RSM proposal beyond reducing computational burden, the proposed controller tuning approach is implemented on a system with a large and highly complex dynamic model. The OMR system is selected due to factors like the wide range of applications [109,110] and the current complexity increment of such tasks [111,112]. This characteristic implies that the OMR system is constantly used in applications facing parametric uncertainties and external disturbances. Therefore, the SIACTA-RSM proposal can be a resourceful strategy to enhance the OMR’s performance.

Due to the SIACTA-RSM proposal being a stochastic controller tuning approach, using descriptive and inferential statistics is an important tool to make formal and general conclusions about the controller tuning behavior. Then, the comparative analysis between the SIACTA-RSM proposal and other well-established controller tuning approaches is the second contribution of the work since it confirms an important computational burden reduction and shows that the SIACTA-RSM achieves the best overall performance in OMRs.

### 1.5. Structure of the Work

The rest of the document has the following structure. Section 2 describes the OMR’s dynamic model and the control system to be tuned by the proposal. Section 3 explains in detail the stages and the optimization algorithm encompassing the SIACTA-RSM proposal in the OMR system. The simulation verification outcomes are presented in Section 4, where statistical comparisons (descriptive and inferential) regarding controller tuning performance and computational burden are discussed, and a graphical comparison related to the evaluated path-tracking problems is offered. Finally, Section 5 discusses and highlights the conclusions of the current work.

## 2. OMR’s Dynamic Model Description

**Figure 1:**
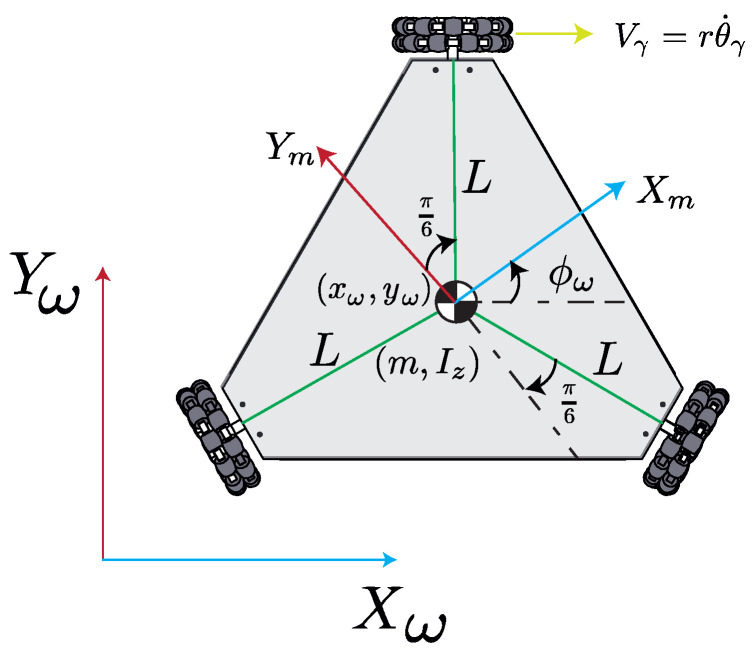
Diagram of OMR.

The OMR’s dynamic model is presented in (1), where $x={\left[x_{1},x_{2},x_{3},x_{4},x_{5},x_{6}\right]}^{T}{=[x_{\omega },y_{\omega },\phi_{\omega },{\dot{x}}_{\omega },{\dot{y}}_{\omega },{\dot{\phi }}_{\omega }]}^{T}\in R^{6}$ is the state vector created from the OMR’s coordinates (positions and orientation) concerning a world inertial frame $\left[X_{\omega },Y_{\omega }\right]$ as depicted in Figure 1. For this model, $x_{\omega }$ and $y_{\omega }$ represent the OMR’s linear positions with respect to the $X_{\omega }$ and $Y_{\omega }$ axes, while $\phi_{\omega }$ stands out for the angular position related to the rotation with respect to the $Z_{\omega }$ axis; hence, ${\dot{x}}_{\omega },\;{\dot{y}}_{\omega }$, and ${\dot{\phi }}_{\omega }$ represent the respective linear and angular velocities. Also, $p={\left[p_{1},p_{2},p_{3},p_{4},p_{5}\right]}^{T}={\left[r,L,m,J,I_{z}\right]}^{T}\in R^{5}$ is the parameter vector whose values and descriptions are presented in Table 2. Similarly, $u=\left[u_{1},u_{2},u_{3}\right]\in R^{3}$ is the OMR’s control signal vector applied to the OMR’s wheels. Moreover, $y\in R^{6}$ is the OMR’s output vector, where $C\in R^{6\times 6}$ is an identity matrix with a diagonal of six elements, where all the states are assumed to be measurable or estimable; therefore, the output vector matches the state vector. Notably, the output vector is assumed to be affected not by the control input vector but only by the state vector. The solution of the differential equation provided by the OMR’s dynamics in (1) is obtained by the Euler method for numerical integration with the initial condition vector $x\left(t_{0}\right)=x_{ini}$. The dynamic model is obtained from [113,114].

(1) $$\begin{array}{c} \dot{x}=f\left(x,p\right)+g\left(x,p\right)u\\ y=Cx \end{array}$$

where

$$f\left(x,p\right)={\left[x_{4},x_{5},x_{6},-3p_{4}\lambda x_{6}x_{5},3p_{4}\lambda x_{4}x_{6},0\right]}^{T}\in R^{6},$$

$$g\left(x,p\right)=\left[\begin{array}{ccc} 0 & 0 & 0\\ 0 & 0 & 0\\ 0 & 0 & 0\\ -\lambda p_{1}\kappa^{-} & 2p_{1}\lambda \sin\left(x_{3}\right) & -\lambda p_{1}\kappa^{+}\\ \lambda p_{1}\eta^{+} & -2p_{1}\lambda \cos\left(x_{3}\right) & \lambda p_{1}\eta^{-}\\ \zeta & \zeta & \zeta \end{array}\right]\in R^{6\times 3},$$

with

$$\begin{array}{cc} \lambda = & \frac{1}{2p_{3}{p_{1}}^{2}+3p_{4}},\;\zeta =\frac{p_{2}p_{1}}{3p_{4}{p_{2}}^{2}+p_{5}{p_{1}}^{2}},\\ \kappa^{\pm }= & \sin\left(x_{3}\right)\pm \sqrt{3}\cos\left(x_{3}\right),\;\eta^{\pm }=\cos\left(x_{3}\right)\pm \sqrt{3}\sin\left(x_{3}\right). \end{array}$$

The control applied to the OMR’s wheels is computed through a proportional derivative (PD) controller as in (2), where $e={\left[e_{1},e_{2},e_{3}\right]}^{T}={\left[x_{d}-x_{1},y_{d}-x_{2},\phi_{d}-x_{3}\right]}^{T}\in R^{3}$ is the error between the desired tracking coordinates and the OMR’s ones and $\dot{e}={\left[\dot{e_{1}},\dot{e_{2}},\dot{e_{3}}\right]}^{T}={\left[{\dot{x}}_{d}-x_{4},{\dot{y}}_{d}-x_{5},{\dot{\phi }}_{d}-x_{6}\right]}^{T}\in R^{3}$ is the error between the desired tracking speeds and the OMR’s ones (linear and angular). Furthermore, $K_{p}=diag\left(k_{1},k_{2},k_{3}\right)\in R^{3\times 3}$ and $K_{D}=diag\left(k_{4},k_{5},k_{6}\right)\in R^{3\times 3}$ are diagonal matrices with the proportional and derivative gains, respectively. Both matrices are conformed by the elements within the controller parameter vector $k={\left[k_{1},\cdots ,k_{6}\right]}^{T}\in R^{6}$. Finally, the OMR’s Jacobian matrix $\overset{˘}{J}\in R^{3\times 3}$ is presented in (3) and is used to relate the OMR’s linear and angular speeds with the OMR’s wheels’ angular speeds.

(2) $$\begin{array}{cc} u & =f_{u}\left(x,k\right)\\ \; & ={\overset{˘}{J}}^{T}\left(K_{p}e+K_{D}\dot{e}\right) \end{array}$$

(3) $$\overset{˘}{J}=\left[\begin{array}{ccc} -\kappa^{-}/3 & 2\sin(x_{3})/3 & -\kappa^{+}/3\\ \kappa^{+}/3 & 2\cos(x_{3})/3 & \kappa^{-}/3\\ p_{2}/3 & p_{2}/3 & p_{2}/3 \end{array}\right]$$

## 3. Development of SIACTA-RSM for OMRs

The surrogate indirect adaptive controller tuning approach (SIACTA) performs two well-defined stages iteratively considering an update frequency $f_{U}=\frac{1}{\Delta T_{U}}$ given by an update period $\Delta T_{U}$. At each update, the adaptive reference model and the controller parameters are computed during the system’s execution. Assuming that the controller tuning process has gathered enough information from the system and reached an update period $\Delta T_{U}$, the first stage consists of a surrogate-assisted identification stage. In this first stage, the information sampled from the system during an identification time span $T_{I}$, before the update period $\Delta T_{U}$, is used to update the adaptive reference model, where in this work, the response surface method (RSM) is employed to acquire the surrogate model. Once the surrogate model is updated, the second stage is the surrogate-based prediction stage. This stage utilizes the new surrogate model to compute the controller’s parameters, aiming to improve the system’s desired performance through bioinspired optimization. The proposed SIACTA then takes advantage of biomimetic elements in the bioinspired optimization and surrogate models to mimic nature’s ability to solve complex problems efficiently, adaptively, and with limited resources.

**Figure 2:**
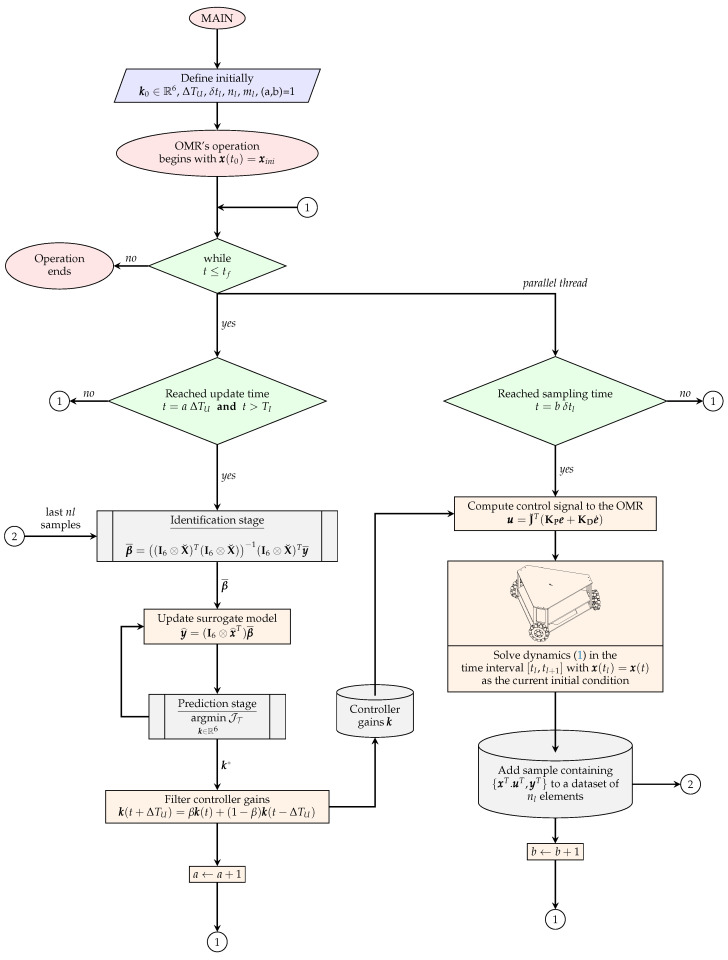
SIACTA-RSM operation scheme to OMRs.

Next, a detailed description of how these stages operate in the surrogate indirect adaptive controller tuning approach based on the response surface method (SIACTA-RSM) applied to the OMR is provided in the next sections. Also, Figure 2 is proportioned to understand the flowchart of the SIACTA-RSM proposal.

### 3.1. Surrogate-Assisted Identification Stage

**Figure 3:**
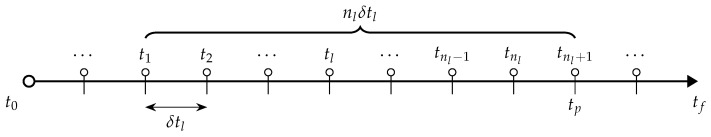
A schematic representation of the time intervals in the identification period.

Let us consider the set of $6n_{l}$ second-degree multivariate polynomials (multiple second-degree multivariate polynomials) [115] groups in $\bar{y}\in R^{6\cdot n_{l}}$ (4) with the use of Kronecker product ⊗ and using the state vector x and the control input vector u as their variables. This expression describes the omnidirectional mobile robot’s behavior in the identification time span $T_{I}=n_{l}\delta t_{l}$ from the identification time window $\left[t_{p}-n_{l}\delta t_{l},t_{p}\right]$ relative to the current time $t_{p}$ with $\delta t_{l}$ as the sampling period, i.e., it contains $n_{l}$ samples of each system’s output, each one related to a polynomial function. It is important to highlight the assumption that the first identification process continues until all system information is obtained, i.e., when the current time $t_{p}$ is greater than or equal to the identification time span ($t_{p}\geq n_{l}\delta t_{l}$). In the first time span of $n_{l}\delta t_{l}$ seconds, the initial controller parameters $k_{0}\in R^{6}$ for the OMR’s control u (2) are set randomly between the chosen bounds in the optimization process or by using another preferred offline controller tuning method (for instance, the method described in [114]). Then, the information of the control signal vector and the state vector from the identification time span is acquired, and the polynomials in (4) can be computed. It is also worth noting that second-degree polynomials are used because the analysis presented in [20,23] shows that further polynomial degrees significantly increase the computational burden but do not enhance the performance achieved by the regression.

(4) $$\bar{y}=\left(I_{6}⊗\overset{˘}{X}\right)\bar{\beta }$$

where the following are true:

- $\bar{y}={\left[y_{1}^{1},\cdots ,y_{1}^{l},\cdots ,y_{1}^{n_{l}},y_{2}^{1},\cdots ,y_{2}^{l},\cdots ,y_{2}^{n_{l}},\cdots ,y_{6}^{1},\cdots ,y_{6}^{l},\cdots ,y_{6}^{n_{l}}\right]}^{T}\in R^{6n_{l}}$ contains the output attributes of the $n_{l}$ instances. This includes the $n_{l}$ samples (instances) for each of the six outputs of the system obtained in the time $t_{l+1}$ using the input attributes in the time $t_{l}$, i.e., the solution of the OMR Equation (1).
- $I_{6}$ is the identity matrix with a size corresponding to the six system outputs.
- $\overset{˘}{X}={\left[{{\overset{˘}{x}}^{1}}^{T},\cdots ,{{\overset{˘}{x}}^{l}}^{T},\cdots ,{{\overset{˘}{x}}^{n_{l}}}^{T}\right]}^{T}\in R^{n_{l}\times 55}$ is a matrix with the variables (input attributes) of the $n_{l}$ second-degree polynomials.
- ${\overset{˘}{x}}^{l}=[1,x_{1}^{l},\cdots ,x_{6}^{l},u_{1}^{l},\cdots ,u_{3}^{l},x_{1}^{l}x_{1}^{l},\cdots ,x_{1}^{l}x_{6}^{l},x_{1}^{l}u_{1}^{l},\cdots ,x_{1}^{l}u_{3}^{l},x_{2}^{l}x_{2}^{l},\cdots ,x_{2}^{l}x_{6}^{l},x_{2}^{l}u_{1}^{l},\cdots ,x_{2}^{l}u_{3}^{l}$, $x_{3}^{l}x_{3}^{l},\cdots ,x_{3}^{l}x_{6}^{l},x_{3}^{l}u_{1}^{l},\cdots ,x_{3}^{l}u_{3}^{l},x_{4}^{l}x_{4}^{l},\cdots ,x_{4}^{l}x_{6}^{l},x_{4}^{l}u_{1}^{l},\cdots ,x_{4}^{l}u_{3}^{l},x_{5}^{l}x_{5}^{l},\cdots ,x_{5}^{l}x_{6}^{l},x_{5}^{l}u_{1}^{l},\cdots ,x_{5}^{l}u_{3}^{l},x_{6}^{l}x_{6}^{l},u_{1}^{l}u_{1}^{l},\cdots ,u_{1}^{l}u_{3}^{l},u_{2}^{l}u_{2}^{l},\cdots ,u_{2}^{l}u_{3}^{l},u_{3}^{l}u_{3}^{l}{]}^{T}={\left[1,{\overset{˘}{x}}_{1}^{l},\cdots ,{\overset{˘}{x}}_{54}^{l}\right]}^{T}\in R^{55}$ contains the variables related to the *l*-th second-degree multivariate polynomial (for example, the *l*-th polynomial model relating the input ${\overset{˘}{x}}^{l}$ and output $y_{1}^{l}$ attributes for the *l*-th instance results in $y_{1}^{l}={\left({\overset{˘}{x}}^{l}\right)}^{T}{\bar{\beta }}_{1}$). The vector includes the *l*-th state and control signal vector sample, $x^{l}$ and $u^{l}$, at the sampling time $t_{l}$. Using the nine variables (state and control input vectors), the total number of terms in the *l*-th polynomial is fifty-five [115]. The schematic diagram of time in Figure 3 shows the related time in the identification time span $T_{I}$.
- $\bar{\beta }={\left[{\bar{\beta }}_{1}^{T},...,{\bar{\beta }}_{6}^{T}\right]}^{T}\in R^{6\cdot 55}$ is the vector that contains the coefficients of the second-degree polynomials for the six outputs of the OMR.

Thus, obtaining $\bar{\beta }$ from (4) through the use of the pseudo inverse of the matrix $\left(I_{6}⊗\overset{˘}{X}\right)\in R^{6\cdot n_{l}\times 6\cdot 55}$ results in (5).

(5) $$\bar{\beta }={\left({\left(I_{6}⊗\overset{˘}{X}\right)}^{T}\left(I_{6}⊗\overset{˘}{X}\right)\right)}^{-1}{\left(I_{6}⊗\overset{˘}{X}\right)}^{T}\bar{y}$$

It is important to assume that the minimum number of samples $n_{l}$ (instances) must be at least the total number of available monomials of the second-degree polynomial grouped in ${\overset{˘}{x}}^{l}$, i.e., $n_{l}\geq 55$ must be fulfilled to ensure a solvable equation system given by $\bar{\beta }$.

Once coefficients of the polynomials $\bar{\beta }$ are computed, they can be used in (6), which provides an approximate behavior $\hat{y}\in R^{6}$ to any posterior input attribute vector $\hat{x}\in R^{55}$ and can predict the robot mobile behavior in the next tuning stage. The input attribute $\hat{x}$ has the same form of ${\overset{˘}{x}}^{l}$.

(6) $$\begin{array}{cc} \hat{y} & =\hat{f}\left(\hat{x},\bar{\beta }\right)\\ \; & =\left(I_{6}⊗{\hat{x}}^{T}\right)\bar{\beta } \end{array}$$

### 3.2. Surrogate-Based Prediction Stage

**Figure 4:**
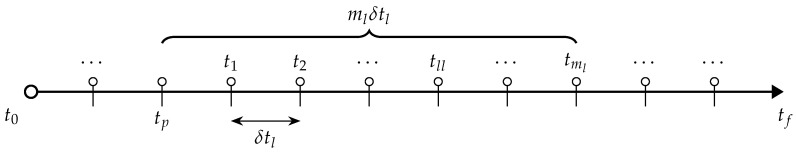
A schematic representation of the time intervals in the prediction period.

Once the surrogate model (6) is obtained, it simulates $m_{l}$ future intervals, each lasting $\delta t_{l}$ seconds, starting from the present time $t_{p}$. When this simulation starts, the current state vector $x(t_{p})$ in the current time $t_{p}$ and the control signal $u(x\left(t_{p}\right),k)$ are considered as the initial conditions in the vector $\hat{x}\left(t_{0}\right)$. The prediction simulation covers a prediction time span $T_{P}$ from the prediction time window $\left[t_{p},t_{p}+m_{l}\delta t_{l}\right]$, as is observed in Figure 4. At each prediction time $t_{ll}$, the surrogate model generates a predictive output vector $\hat{y}\left(t_{ll+1}\right)=\hat{f}\left(\hat{x}\left(t_{ll}\right),\bar{\beta }\right)$ (6), representing the OMR behavior. Consequently, the subsequent vector $\hat{x}\left(t_{ll}\right)$ comprises the predicted vector $\hat{y}\left(t_{ll}\right)$ and the computed control signal $u(\hat{y}\left(t_{ll}\right),k)$, which is determined using (2). The vector $\hat{x}\left(t_{ll}\right)$ is then used to compute the next predictive output vector $\hat{y}\left(t_{ll+1}\right)$. The predictive output vector estimates the system states over the prediction time span.

Through this prediction stage, it is possible to simulate and correct the behavior that the OMR has presented. In this work, the desired behavior is related to reducing the integral squared error (ISE) of the path-tracking problem in (7), where ${\hat{e}}_{1}=x_{d}-{\hat{y}}_{1}$, ${\hat{e}}_{2}=y_{d}-{\hat{y}}_{2}$, and ${\hat{e}}_{3}=\phi_{d}-{\hat{y}}_{3}$ are the errors between the desired and estimated positions (linear and angular). The angular error ${\hat{e}}_{3}$ is transformed into linear measurements, multiplying it by the wheels’ distance to the mass center in (7).

(7) $$J_{T}=\int_{t\in [t_{p},t_{p}+m_{l}\delta t_{l}]}\left({{\hat{e}}_{1}}^{2}+{{\hat{e}}_{2}}^{2}+{p_{2}}^{2}{{\hat{e}}_{3}}^{2}\right)dt$$

Consequently, the optimization problem to be solved during this stage is shown in (8), where $k_{min}={\left[k_{min,1},\cdots ,k_{min,6}\right]}^{T}\in R^{6}$ and $k_{max}={\left[k_{max,1},\cdots ,k_{max,6}\right]}^{T}\in R^{6}$ are the optimization problem’s lower and upper boundaries, respectively, for the controller proportional and derivative gains. Notably, the problem is subject to the surrogate model instead of being related to the OMR dynamic model. An important clarification is that a mono-objective approach is selected rather than a multi-objective one, considering that the latter requires a decision-making process such as those used in [116,117], which means additional computational burden and tuning of the decision-maker. Therefore, the more straightforward first research step is to use a mono-objective problem.

(8) $$\begin{array}{cc} \; & \min_{k\in R^{6}}J_{T}\\ s.t & \left\{\begin{array}{c} \hat{y}=\hat{f}\left(\hat{x},\bar{\beta }\right)\\ k_{min}\leq k\leq k_{max} \end{array}\right. \end{array}$$

Finally, since this iterative process aims to deal with stochastic parametric uncertainties and external disturbances, the controller parameters k might change abruptly. These rapid changes can produce undesired oscillations in the system. Hence, a discrete-time implementation of a Resistor–Capacitor (RC) low-pass filter given by $k\left(t+\Delta T_{U}\right)=\beta k\left(t\right)+\left(1-\beta \right)k\left(t-\Delta T_{U}\right)$ is applied to k as in [118], where the smoothing factor is set as β=0.5. This filter reduces undesired oscillations in the system’s performance by averaging the new and last computed controller parameters. Notably, the proposal operation requires an initial controller parameter vector $k_{0}\in R^{6}$ to be used until the first update interval is reached. This initial controller parameter vector $k_{0}$ can be set by any other offline controller tuning approach or even randomly.

### 3.3. Optimizer

It is important to note that the controller tuning optimization problem (8) includes the highly nonlinear behaviors of the OMR’s dynamic model presented in (1). Besides the high nonlinearities, the controller tuning optimization problem (8) could present discontinuities due to the tracked trajectory, where it is not easy to guess an initial solution at each update instant. Therefore, it cannot be solved analytically, and the classical optimization methods are not likely to find suitable solutions. In this case, population-based bioinspired algorithms are potential alternatives to solve the controller tuning problem. These bioinspired algorithms are not limited to only continuous functions. Also, they only require a search region instead of an initial search point [119]. Particularly, the differential evolution algorithm in its online version (ODE) has proved to be a reliable tool in online controller tuning approaches as presented in [20,23,120], where the online version consists of including the best solution of a previous search within the initial population of a current search, enhancing the initial exploration phase. The exact DE variant in ODE is the best/1/bin, which has been used in the online controller tuning approach [20,120]. The description of the implemented version of ODE is presented in Algorithm 1. For this algorithm, NP and $G_{max}$ are the population size and the maximum generations considered, respectively. Also, the operation to create a new individual is presented in code line ten, where Cr is the crossover rate that determines the population crossover probability and $\left[F_{min},F_{max}\right]$ is the mutation range from where the mutation factor *F* is selected randomly at each generation, as presented in code line seven. Finally, in code line three, it is observed that the result from the previous update interval is included in the initial population of the new search.
**Algorithm 1:** ODE pseudocode**Inputs:**
▹ Result from previous update interval ($k_{prev}$), ▹ Maximum generations ($G_{max}$), ▹ Population size (NP), ▹ Boundaries range ($\left[k_{min},k_{max}\right]$), ▹ Crossover rate (Cr), ▹ Mutation factor range ($\left[F_{min},F_{max}\right]$), ▹ Objective funtion (OF).
**Output:** ◃ Best solution $k^{*}$.
    1: G←1.
    2: Randomly create an initial population $K^{G}$ of (NP−1) individuals, where each individual $k\in [k_{min},k_{max}]$.
    3: Include $k_{prev}\in K^{G}$.
    4: Evaluate the objective function (OF) (7) of the individuals in $K^{G}$.
    5: Obtain the best individual $k_{best}^{G}$ from $K^{G}$.
    6: **while** $G<G_{max}$ 
**do**
    7:    Randomly obtain mutation factor $F\in [F_{min},F_{max}]$. 
    8:    **for each**  $k_{i}^{G}\in K^{G}$ **do**

    9:        Randomly obtain two individuals $k_{r_{1}}$ and $k_{r_{2}}$ from $K^{G}$, where $k_{i}\neq k_{r_{1}}\neq k_{r_{2}}$. 
  10:        Generate the j−th terms of the individual $k_{new}$∀j=1,⋯,6:               $k_{new,j}=\left\{\begin{array}{cc} k_{best,j}^{G}+F\left(k_{r_{1},j}^{G}-k_{r_{2},j}^{G}\right)\;, & \mathrm{if}\;rnd\left(0,1\right)<CR\vee j_{rnd}=j\\ k_{i,j}^{G}\;, & \mathrm{otherwise} \end{array}\right.$.
  11:        Obtain the OF (7) of $k_{new}$.
  12:        Select the one to pass to $K^{G+1}$ between $k_{new}$ and $k_{i}^{G}$, based on their OF (7).
  13:    **end for**
  14:    G←G+1.
  15:    Obtain the best individual $k_{best}^{G}$ from $K^{G}$.
  16: **end while**
  17: $k^{*}\leftarrow k_{best}^{G}$.   18: **return** $k^{*}$.

It is important to point out that other optimizers can be implemented, with the only consideration that the complete SIACTA-RSM operation must compute the controller parameters within the update interval $\Delta T_{U}$, as shown in the flowchart of the SIACTA-RSM proposal in Figure 2. Furthermore, it is essential to remember that the proposal starts to operate once $n_{l}$ samples have been gathered, which are required to carry out the identification stage. Therefore, during the beginning of the system operation ($t<n_{l}\delta t_{l}$), the initial controller parameter vector $k_{0}\in R^{6}$ is set by any other method, as described previously. Once the condition of the first $n_{l}$ samples is fulfilled, the proposal starts its iterative process, and this is repeated at each $\Delta T_{U}$ instant. Then, the proposal obtains the controller parameters to compensate for any unconsidered adverse condition.

## 4. Simulation Verification

In this section, various experiments conducted in a simulation environment are developed to systematically investigate and validate the performance of the proposed SIACTA-RSM. These experiments consider uncertainties and perturbations like those presented in a real-world scenario.

### 4.1. Description of Controller Tuning Approaches to Be Compared

Besides the surrogate indirect adaptive controller tuning approach based on the response surface method (SIACTA-RSM) proposal for the OMR’s controller tuning, three additional controller tuning approaches are selected to make a comparative analysis. The first one is related to the online controller tuning approach. The indirect adaptive controller tuning approach based on the generalized dynamic model (IACTA-GDM) [120] is used for this purpose and to test the SIACTA-RSM proposal improvements regarding the computational burden related to the update process, illustrating the possible advantages or drawbacks of the inclusion of the approximated model in the proposed surrogate tuning approach. On the other hand, the second tuning approach is an offline one, where the Robust Tuning Approach for Controller Gains (RTACG) [114] is used. It is included to analyze the robustness of the proposal under parametric uncertainties. Finally, the third comparative controller tuning approach is offline, where the Non-Robust Tuning Approach for Controller Gains (N-RTACG) [113] is selected to observe the behavior of the proposal under ideal conditions. These three controller tuning approaches will enlighten the characteristics of the SIACTA-RSM proposal in different aspects, like behavior without parametric uncertainties nor disturbances, robustness analysis under parametric uncertainties, and computational burden related to the controller parameters updating. Notably, the three controller tuning approaches are examples of cutting-edge research performed within the last five years, each one with its own perks and drawbacks, helping to analyze the diverse criteria of the SIACTA-RSM proposal.

The conditions of the controller tuning approaches are as follows:

- Considering that the N-RTACG and RTACG are offline tuning approaches, their controller parameter vectors k are fixed values during the execution. The N-RTACG controller parameter vector is kN=[1829.972,4937.868,2578.833,24.999,24.999,15.898]T [113], and that of the RTACG is kR=[1483.5170,1483.4498,2637.6842,68.0400,67.9976,16.1031]T [114]. Notably, these controller tuning approaches were obtained for exactly the same system tested in the current work. Additionally, these approaches implemented the same bioinspired optimization algorithm (without the surrogate model adaptation) for the same objective function and considered an exhaustive empirical analysis to select the best solution. These procedures increase the fairness of the comparison.
- Since IACTA-GDM and the SIACTA-RSM are online controller tuning approaches, they share the same stages (identification and prediction). In the case of SIACTA-RSM, a surrogate model is included, rather than the generalized dynamic model, as in IACTA-GDM, reducing the computational burden. Hence, looking for a fair comparison, both share the following parameters:
  – Regarding the update and sampling frequencies used in the online controller tuning approaches, the controller parameter vector is updated at each update period $\Delta T_{U}=5$ (ms), where this value has been applied successfully dealing with parametric uncertainties in other electromechanical systems in the real-time implementation of the IACTA [11,121]. Also, the sampling period is $\delta t_{l}=5$ (ms), since it allows the acquisition of the data of the OMR’s dynamics in a real platform [113,114].
  – In the identification stage process, $n_{l}=100$ previous intervals are considered, defining an identification time span of $T_{I}=500$ (ms) [20]. On the other hand, the prediction stage process uses $m_{l}=5$ posterior intervals, forming a prediction time span of $T_{P}=25$ (ms) [20]. The initial controller parameter vector $k_{0}$ is set randomly within the search region of the bioinspired optimizer algorithm ($k_{min}\;\leq \;k_{0}\;\leq \;k_{max}$). This region is set in the following point.
  – Regarding the optimizer employed, the ODE algorithm uses a population size of NP=20 individuals, a maximum number of generations of $G_{max}=20$, a mutation range of F=[0.3,0.9], and a crossover rate of $C_{r}=0.6$. The values are obtained from [20], where they are applied in the SIACTA-RSM and IACTA. Moreover, the upper and lower boundaries ($k_{max}$ and $k_{min}$, respectively) for the search region of the controller tuning optimization problem (8) are established through N-RTACG and RTACG controller parameter vectors (kN and kR, respectively) as $k_{max,i}=1.2\max{(}^{N}k_{i}{,}^{R}k_{i})$ and $k_{min,i}=0.8\min{(}^{N}k_{i}{,}^{R}k_{i})$, ∀i∈{1,⋯,6}. Thus, the resulting lower and upper boundaries are $k_{min}={\left[1186.8136,1186.75984,2063.0664,19.9992,19.9992,12.7184\right]}^{T}\in R^{6}$ and $k_{max}={\left[2195.9664,5925.4416,3165.2210,81.6480,81.5971,19.3237\right]}^{T}\in R^{6}$. These boundaries are set considering that the offline controller tuning approaches offer a region unaffected by the OMR’s high nonlinearities instead of finding a region through an exhaustive (computational and time-wasting) empirical strategy. Additionally, it is worth pointing out that the initial controller parameter vector is created randomly between these boundaries until the first identification period is completed.

### 4.2. Simulation Conditions

The aforementioned controller tuning approaches are tested in two different path-tracking problems, where thirty independent executions are carried out in each experiment per controller tuning approach. Before starting with the description of the experiments, it is worth pointing out that the simulations are carried out on MATLAB R2022b in a computer equipped with an ${\mathrm{Intel}}^{®}{\mathrm{Core}}^{\mathrm{TM}}$ i9-13900HX CPU running at a clock speed of 3.64 GHz and 32 GB of RAM.

The first experiment considers ideal conditions without parametric uncertainties or external disturbances. The first path-tracking problem is a hypocycloid path whose parametric details are presented in the first row of Table 3. The path-tracking problem must be solved in a time $t_{f}=120$ (s). Notably, the first experiment presents the same conditions used by the N-RTACG and RTACG [113,114].

On the other hand, aiming to test the controller tuning approaches under hard conditions, the second experiment considers a different path (Lissajous path), whose parametric details are presented in the second row of Table 3. The path-tracking problem must be solved at $t_{f}=60$ (s). Beyond the path modification and the velocity increment, the second experiment considers continuous variations in the OMR’s parameter vector $p={\left[p_{1},p_{2},p_{3},p_{4},p_{5}\right]}^{T}={\left[r,L,m,J,I_{z}\right]}^{T}\in R^{5}$ as in (9), where Fourier expansions of third order create quasi-square waves, where $\eta \left(t\right)=\frac{2}{\pi }\left(\sum_{n=1}^{3}\frac{n}{2n-1}\sin\left(2\pi \left(2n-1\right)f\left(t+t_{off}\right)\right)\right)$ and $\rho \left(t\right)=\frac{2}{\pi }\left(\sum_{n=1}^{3}\frac{n}{2n-1}\sin\left(2\pi \left(2n-1\right)ft\right)\right)$ are the change ratio functions for cosine and sine approximations, respectively, considering a frequency $f=\frac{1}{t_{f}}$ and an offset $t_{off}=\frac{t_{f}}{4}$. Also, the second experiment considers continuous variation in the control vector $u={\left[u_{1},u_{2},u_{3}\right]}^{T}\in R^{3}$ as in (10), following the same quasi-square signals strategy. Moreover, the second experiment considers friction with a fixed constant $f_{\mu }=1.0$ and random Gaussian noise applied to the linear and angular speeds within an interval [−5 × 10^−3^, 5 × 10^−3^] (the worst condition tested by the RTACG [114]).

(9) $$p_{i}=\left\{\begin{array}{cc} p_{i}+0.2p_{i}\left(\eta (t)\right), & \mathrm{if}\;i=1\\ p_{i}+0.2p_{i}\left(\rho (t)\right), & \mathrm{if}\;i=2\\ 10p_{i}+4p_{i}\left(\eta (t)\right), & \mathrm{if}\;i=3\\ 2p_{i}+0.9p_{i}\left(\rho (t)\right), & \mathrm{if}\;i=4\\ p_{i}+0.3p_{i}\left(\eta (t)\right), & \mathrm{if}\;i=5 \end{array}\right.$$

(10) $$u_{i}=\left\{\begin{array}{cc} u_{i}+0.2u_{i}\left(\eta (t)\right), & \mathrm{if}\;i\;\mathrm{is}\;\mathrm{odd}\\ u_{i}+0.2u_{i}\left(\rho (t)\right), & \mathrm{otherwise} \end{array}\right.,\;\forall \;i\in \left\{1,2,3\right\}$$

### 4.3. Analysis Methodology

The mentioned experiments are analyzed through three guidelines. The first guideline encompasses graphical comparisons to proportionate a general overview of the path-tracking results. The second guideline evaluates the OMR system’s operation through two criteria: the first one is the controller tuning performance measured via the controller tuning functional $J_{T}$ (7), and the second one is the Integral Square of the Control Signal (ISU) applied to the wheels of the OMR given by $ISU=\int_{t_{0}}^{t_{f}}u^{T}u\;dt$.

The third guideline performs a computational burden analysis by evaluating the average time required to compute an update interval $\Delta T_{U}$ during a complete execution, hereinafter referred to as “solver time” $S_{T}$. Finally, a general discussion of the results is carried out by evaluating the normalized trade-off $T_{O}$ (11) between the solver time and the controller tuning performance, where $\hat{J_{T}}$ and $\hat{J_{T}}$ represent the normalized values between 0 and 1 of each criterion, respectively. The trade-off $T_{O}$ is assessed by measuring the normalized distance to the ideal point (minimum criteria values) across thirty executions.

(11) $$TO_{i}=\sqrt{\left({\hat{J_{T}}}_{i}^{2}\right)+\left({\hat{S_{T}}}_{i}^{2}\right)}\;,\;\;\forall \;i\in \left\{1,\cdots ,30\right\}$$

The main focus of this last discussion is to find the best trade-off related to the solution nearest to the Pareto front’s knee-point. A lower value of $T_{O}$ is preferred, indicating a better trade-off between the solver time $S_{T}$ and the controller tuning performance $J_{T}$. Notably, the evaluation of the third guideline applies only to the online controller tuning approaches (SIACTA-RSM and IACTA-GDM), as they are the only methods that iteratively update the controller parameters.

### 4.4. Simulation Results

#### 4.4.1. Graphical Comparisons

**Figure 5:**
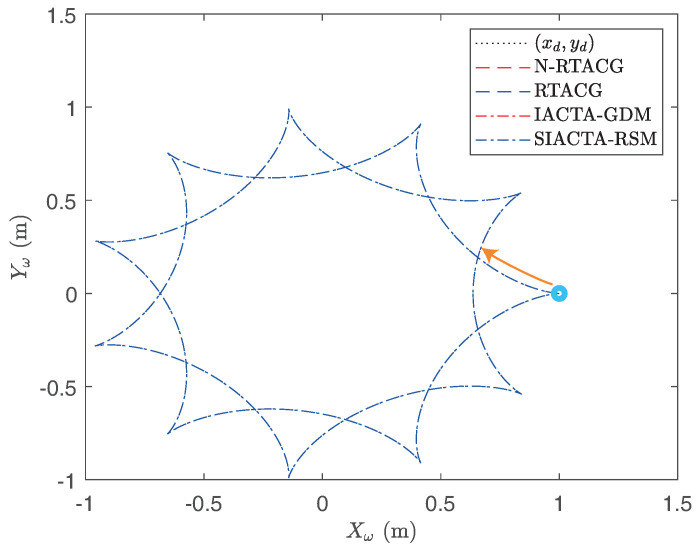
Path-tracking results: Experiment 1 with the OMR’s initial condition $x\left(t_{0}\right)={\left[1,0,0,0,0,0\right]}^{T}$.

**Figure 6:**
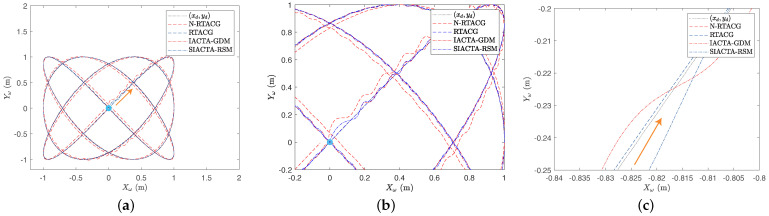
Path-tracking results: Experiment 2 with the OMR’s initial condition $x\left(t_{0}\right)={\left[0,0,0,0,0,0\right]}^{T}$. (**a**) General path-tracking results. (**b**) Close-up view 1. (**c**) Close-up view 2.

Aiming for a fast overview of the behavior of the implemented controller tuning approaches, a graphical comparison is carried out. Figure 5 and Figure 6 depict the path-tracking results for the hypocycloid and Lisaajous paths, respectively, where a blue circle depicts the initial point of the track, and an orange arrow presents the initial trajectory route. Regarding the first experiment, it is observed in Figure 5 that it is difficult to distinguish between the N-RTACG, RTACG, IACTA-GDM, and SIACTA-RSM since they present visually similar behaviors. This is because the first experiment considers ideal conditions without parametric uncertainties nor external disturbances. However, a statistical analysis of outcomes, highlighting the differences among the controller tuning approaches, is presented in the next section.

Concerning the second experiment, it can be seen in Figure 6a that the N-RTACG stands out as the worst controller tuning approach, unable to follow the Lissajous path. On the other hand, the RTACG, IACTA-GDM, and SIACTA-RSM present similar behaviors. Figure 6b presents a close-up view of the beginning of the tracking problem, where it is observed that during the start-up of the system, the IACTA-GDM and SIACTA-RSM present significant oscillations, which are the effect of the random selection of the initial controller gain vector. However, these oscillations are reduced once the update intervals begin. Moreover, Figure 6c displays a close-up view of another region of interest, where the difference between the online controller tuning approaches and the RTACG is more evident. In this region, it is observed that even if the RTACG presents fewer oscillations, it cannot reach the desired path moving parallel to it.

Next, a statistical comparison is conducted to understand the difference among the controller tuning approaches implemented measurably.

#### 4.4.2. System Operation Comparison

**Figure 7:**
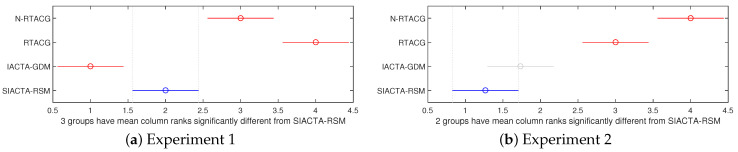
Bonferroni post hoc pairwise comparison regarding $J_{T}$.

Table 4 depicts the statistical results (descriptive and inferential) regarding the controller tuning functional $J_{T}$ related to the path-tracking error reported by the controller tuning approaches. The rows are grouped into two sets, corresponding to the statistical outcomes of the first and second experiments, respectively, where each set follows the next structure. The second column presents the names of the compared controller tuning approaches. The best, worst, mean, and standard deviation of the thirty executions per controller tuning approach are reported from the third to the sixth columns, respectively, where the best reported outcomes per column are noted in boldface. The seventh column presents the obtained mean Friedman ranks. The eighth column depicts the *p*-value obtained by the multi-comparative Friedman test, where if the *p*-value is lower than a statistical significance α=0.05, i.e., *p*-value <α, it indicates that the null hypothesis $h_{0}$ is rejected, implying that at least one of the controller tuning approaches is statistically different from the rest [122]. Since the *p*-values reported in Table 4 reject the null hypothesis $h_{0}$, indicating that at least one of the controller tuning approaches per experiment is statistically different from the rest, post hoc pairwise comparisons with a Bonferroni correction are carried out [122]. Figure 7a,b display the results of the post hoc pairwise comparisons, where the horizontal lines indicate the mean rank distributions of the thirty executions per controller tuning approach. When lines overlap, the horizontal line is gray, indicating the absence of a significant difference between the algorithms (i.e., the *p*-values in those comparisons exceed the chosen significance level), and no inference about the data distributions can be made. In contrast, algorithms that differ considerably from one to another have no overlap in their current lines; this indicates that the *p*-value is below the chosen significance level. Based on rankings, the algorithm further to the left in Figure 7a,b wins (it presents the lowest rank value). The horizontal blue line distribution indicates the element of interest in the comparative tests (SIACTA-RSM proposal). The horizontal red line indicates the other groups of elements (N-RTACG, RTACG, and IACTA-GDM), which are significantly different.

The following highlights from the outcomes regarding the controller tuning function are presented:

- In the first experiment, the general conclusion of the controller tuning comparison, given by the inferential statistical outcomes, shows that without uncertainties and perturbations, the SIACTA-RSM is better regarding controller tuning performance $J_{T}$ than the offline controller tuning approaches N-RTACG and RTACG, but the SIACTA-RSM is worse than the IACTA-GDM. This also indicates that the online controller approaches achieve better mean outcomes of the thirty executions than the offline controller tuning approaches.
- In particular, based on the descriptive statistic results of the first experiment, the IACTA-GDM diminishes by 39.54% the outcomes of the SIACTA-RSM. Nonetheless, the SIACTA-RSM yields a better standard deviation, being thirty-five times lower than the IACTA-GDM’s, meaning it is a more confident (precise) approach. Regarding the SIACTA-RSM against the offline controller tuning approaches, it is observed that the SIACTA-RSM reduces by 18.94% the N-RTACG’s mean result and by 19.24% the RTACG’s.
- In the second experiment, when parametric uncertainties and external disturbances are applied to the closed-loop system, it is statistically confirmed that the online controller tuning approaches (SIACTA-RSM and IACTA-GDM) are superior, regarding the controller tuning performance $J_{T}$, to the offline controller tuning approaches (N-RTACG and RTACG). Specifically, the SIACTA-RSM proposal significantly reduces the RTACG’s mean result by 93%.
- Both the SIACTA-RSM and IACTA-GDM do not present significant differences in the inferential statistic, meaning a winner cannot be stated between them. Nevertheless, the particular results, given by the descriptive statistics in Table 4, show that the SIACTA-RSM proposal reduces by 1.52% the mean result of IACTA-GDM in its thirty executions, and it has the lower (better) mean rank (see Figure 7b), implying that, with these particular data, the SIACTA-RSM proposal remains a trustworthy approach.

**Figure 8:**
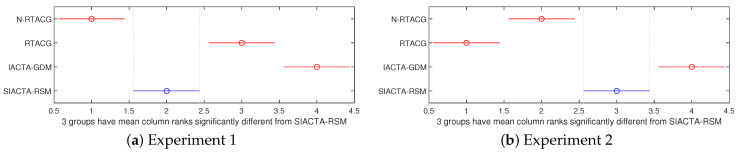
Bonferroni post hoc pairwise comparison regarding ISU.

Following a similar structure to the above results, Table 5 (descriptive statistics and multi-comparative test per experiment) and Figure 8a,b (post hoc pairwise comparisons with Bonferroni correction) are proportioned, whose information is related to the Integral Square of the Control Signal (ISU) applied to the OMR’s wheels. The highlights are as follows:

- In the absence of parametric uncertainties and external disturbances in the first experiment, the N-RATCG approach stands out as the controller tuning approach with a lower ISU, followed by the SIACTA-RSM, RTACG, and IACTA-GDM, in that order. In particular, it reduces by a negligible 0.013% the SIACTA-RSM’s mean outcome (second-best reported ISU). Similarly, the SIACTA-RSM decreases (it is better) by 0.019% the RTACG’s mean results in the first experiment. The RTACG approach not only has worse controller tuning performance (based on Figure 7a) than the SIACTA-RSM but also requires a greater ISU (based on Figure 8b) to achieve the path-tracking task.
- According to the first experiment’s results, it is observed that the SIACTA-RSM enhances the IACTA-GDM’s mean result in the ISU by 5.24%. This result correlates with controller performance $J_{T}$ since the IACTA-GDM presents better controller behavior, which causes it to use greater torque to execute its path-tracking function.
- Under parametric uncertainties and external disturbances applied in the second experiment, the RATCG approach possesses the best results regarding the ISU, showing the benefits of its robustness strategies, followed by the N-RTACG, SIACTA-RSM, and IACTA-GDM, in that order. Instead of reaching the best behavior for a particular task, the RATCG aims to achieve a generalized behavior under different conditions. In particular, the RTACG reduces by 99.11% the SIACTA-RSM’s mean results regarding the ISU (applied torque) in the OMR’s wheels. This outcome is similar to the SIACTA-RSM’s advantage over the RTACG regarding the controller tuning performance $J_{T}$ (as presented in Table 4), showing a correlation between criteria, i.e., a better ISU means a worse $J_{T}$, and vice versa.
- Regarding the online controller tuning approaches, it is observed that the SIACTA-RSM outperforms the ISU value of the IACTA-GDM in the second experiment. In particular, the SIACTA-RSM reduces by 21.78% the IACTA-GDM’s ISU mean result. Considering this result and the SIACTA-RSM’s similar performance over IACTA-GDM, regarding the controller tuning functional $J_{T}$, it is clear that the SIACTA-RSM boosts the OMR’s functionality under adverse conditions beyond the IACTA-GDM’s capabilities.

#### 4.4.3. Computational Burden Analysis of the Online Controller Tuning Approaches

Considering that online controller tuning approaches are the only ones with high computational requirements related to their periodic controller parameter update intervals, the SIACTA-RSM and IACTA-GDM approaches are the only ones whose computational burdens are analyzed. Furthermore, since the computational burden is related to the controller tuning approach rather than the OMR system’s condition, only the second experiment’s solver time $S_{T}$ outcomes are evaluated. Table 6 displays the statistical results (descriptive and inferential) regarding the solver time $S_{T}$. The second column presents the names of online controller tuning approaches. The best, worst, mean, and standard deviation of thirty executions per controller tuning approach are presented from the third to the sixth columns, respectively, where the best obtained outcomes per column are registered in **boldface**. The symbols in the seventh column (+,−,≈) indicate superiority, inferiority, or similarity, respectively. These represent the results of the Wilcoxon signed-rank test, whose *p*-value is reported in the eighth column, where if the *p*-value is lower than a statistical significance α=0.05, i.e., *p*-value <α, it indicates that the null hypothesis $h_{0}$ is rejected, meaning that the compared approaches are statistically different. The outcomes (descriptive and inferential) show that the SIACTA-RSM is superior regarding solver time $S_{T}$, significantly reducing by 60.11% the IACTA-GDM’s mean solver time.

### 4.5. Results Discussion

Considering the results obtained through the previous experiments, it is observed that the online controller tuning approaches (SIACTA-RSM and IACTA-GDM) considerably surpass the controller tuning performance $J_{T}$ obtained in both experiments with respect to the offline approaches (N-RTACG and RTACG). This implies that the online controller tuning approaches are better at dealing with parametric uncertainties and external disturbances. In particular, the SIACTA-RSM proposal improves by 18.94% the N-RTACG’s mean result in the first experiment and by an outstanding 93% the RTACG’s mean result in the second one.

By analyzing the online controller tuning approaches in the first experiment, it is observed that under ideal conditions (without parametric uncertainties or external disturbances), the SIACTA-RSM proposal decreases the controller tuning performance $J_{T}$ by 39.54% with respect to the IACTA-GDM, boosts the solver time performance $S_{T}$ by 60.11%, and reduces the applied torque ISU by 5.4%. Yet, under the presence of complex parametric uncertainties and external disturbances in the second experiment, the SIACTA-RSM proposal presents a similar controller tuning performance $J_{T}$ with respect to IACTA-GDM. However, SIACTA-RSM reduces the computational burden regarding the solver time $S_{T}$ by 62.85% and the applied torque by 21.78% in terms of ISU.

A deep analysis of the normalized trade-off between the achieved controller tuning performance $J_{T}$ and the employed solver time $S_{T}$ is carried out to provide an insight into the synergetic behavior of the proposal. The trade-off $T_{O}$ is accomplished via (11), and Table 7 provides the results. Following a similar structure, Table 7 presents the statistical results (descriptive and inferential) related to the trade-off $T_{O}$ for both experiments in two groups of rows, following the structure used in Table 6. The SIACTA-RSM proposal yields the best trade-off in both experiments according to the Wilcoxon signed-rank test. In particular, SIACTA-RSM reduces by 3.92% the IACTA-GDM’s mean $T_{O}$ results in the first experiment. Moreover, the SIACTA-RSM proposal significantly increases its superiority regarding the second experiment’s $T_{O}$ results, diminishing by 62.04% the IACTA-GDM’s outcomes. The superiority in both experiments makes the SIACTA-RSM proposal stand out as the best controller tuning approach, offering the best trade-off between solver time and controller tuning performance.

Considering the outcomes obtained in the comparisons between the SIACTA-RSM proposal and the other controller tuning approaches implemented, it is evident that the SIACTA-RSM for omnidirectional mobile robots presents more benefits in a real scenario, where parametric uncertainties and external disturbances are unavoidable.

### 4.6. Comparison Against Other Surrogate Model-Based Bioinspired Optimization

Aiming to widen the comparison scope and highlight the advantages of the current proposal, the developed SIACTA-RSM approach is tested against another popular surrogate modeling strategy. Gaussian process regression (GPR) is selected as the surrogate model competitor considering its wide popularity in implementations together with bioinspired optimization algorithms, as observed in [123,124,125,126,127,128,129]. The competitor is referred to as the surrogate indirect adaptive controller tuning approach based on Gaussian process regression (SIACTA-GPR). Both approaches are tested on thirty executions in a Lemsnicata path whose parametric description is presented in Table 8. Moreover, the simulations also include the noise signals of (9) and (10) for the parameters and control signal vectors, respectively. Furthermore, it also considers friction with a fixed constant $f_{\mu }=1.0$ and random Gaussian noise applied to the linear and angular speeds within an interval [−5 × 10^−3^, 5 × 10^−3^]. It is worth pointing out that both surrogate indirect adaptive controller tuning approaches implemented the same sampling interval $\delta t_{l}=5$ (ms), update interval $\Delta T_{U}=5$ (ms), previous samples $n_{l}=100$, and posterior samples $m_{l}=5$ as the previous comparisons. Similarly, the same conditions for the ODE algorithm are taken into account. Those conditions aim to increase the fairness of the comparison.

**Figure 9:**
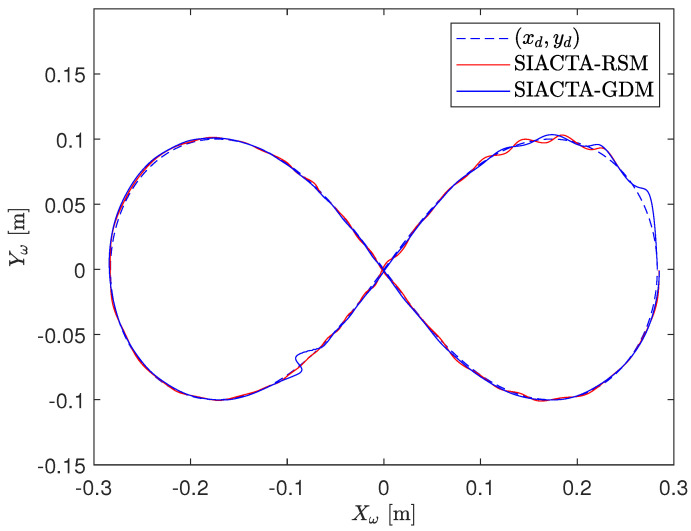
Lemniscate achieved paths with OMR’s initial condition $x\left(t_{0}\right)={\left[0.2828,0,0,0,0,0\right]}^{T}$.

Figure 9 displays a graphical overview of the achieved lemniscate paths, presenting the closest to the mean execution of each controller tuning approach. It is observed that the SIACTA-RSM proposal presents shorter oscillations than the popular SIACTA-GPR, achieving better stability during OMR operation. To better understand this difference, a statistical analysis is presented.

Table 9 displays the outcomes obtained by SIACTA-RSM and SIACTA-GP. Table 9 is organized in three sets of rows, each one to display the outcomes related to the controller tuning objective functional $J_{T}$ (7), the solver time $J_{T}$, and the normalized trade-off between them $T_{O}$ (11), according to the header on the first column. The second column displays the respective controller approach in the pairwise comparison on each set. The best, worst, mean, and standard deviation results of the thirty executions are displayed from the third to the sixth columns. The seventh and eighth columns depict the outcomes of a Wilcoxon signed-rank test. If the eighth column reports a *p*-value lower than the statistical significance α=0.05, i.e., p-value<0.05, it means that the null hypothesis $h_{0}$ is rejected, implying that one of the two controller tuning approaches is statistically different from the other. If the null hypothesis is rejected, the symbols in the seventh column (+, −, ≈) indicate superiority, inferiority, or similarity of the respective controller tuning approach evaluated.

It is observed that the SIACTA-RSM proposal outperforms the popular SIACTA-GPR approach in all the evaluated criteria. The SIACTA-RSM proposal decreases by 63.31%, 91.37%, and 72.89% the mean outcomes of SIACTA-GPR related to $J_{T}$, $S_{T}$, and $T_{O}$, respectively. The SIACTA-RSM proposal stands out as the best surrogate indirect adaptive controller tuning approach for the omnidirectional mobile robot despite SIACTA-GPR’s simplicity and popularity. Together with the state-of-the-art review presented here, these outcomes corroborate the surrogate model-based optimization algorithms, an interesting and potential research field, especially in control applications.

## 5. Conclusions

This work presents the surrogate indirect adaptive controller tuning approach based on the response surface method (SIACTA-RSM) for omnidirectional mobile robots. The proposal employs a multivariate-multitarget polynomial regressor to dynamically compute the reference model and update the controller parameters, enhancing the OMR’s performance during task execution while reducing the computational time required to solve such update processes. The proposal’s competitiveness is evaluated against three cutting-edge techniques (IACTA-GDM, N-RTACG, and RTACG).

The findings of comparative tests show that the online controller tuning approaches (SIACTA-RSM and IACTA-GDM) significantly outperform the offline ones (N-RTACG and RTACG) in terms of controller tuning performance.

After analyzing online controller tuning approaches in a realistic environment with parametric uncertainties and external disturbances, the descriptive and inferential statistics indicate that it is not possible to differentiate the controller tuning performance, concluding that both provide similar outcomes. Nevertheless, the SIACTA-RSM proposal for OMRs presents a significant reduction of 62.85% in the computation time to update the controller gains. Therefore, the results presented in this work underscore the SIACTA-RSM proposal as the most effective controller tuning approach for handling parametric uncertainties and perturbations. This is due to its well-balanced synergy between controller performance and computation time for updating controller parameters. The proposal also reduces the energy consumption by 5.4% (related to the applied torque). Furthermore, a posterior comparison is conducted against another surrogate indirect adaptive controller tuning approach based on Gaussian process regression (SIACTA-GPR). In such a comparison based on thirty executions, the SIACTA-RSM proposal outperforms the SIACTA-GPR by 63.31% in its mean performance while significantly reducing the computational burden by up to 91%.

The control performance, computation time, and energy consumption characteristics achieved by the SIACTA-RSM proposal make it the OMR’s most efficient controller tuning approach. Implementing biomimetic elements in the bioinspired algorithm and the response surface method offers a noteworthy capacity to deal with parametric uncertainties online, overcoming the limits in the required computational resources of the traditional indirect adaptive controller tuning approach. These properties make the SIACTA-RSM proposal a promising tool to be incorporated into embedded systems of autonomous omnidirectional mobile robots. Furthermore, the comparison with respect to another surrogate modeling strategy (Gaussian process regression) highlighted the relevance of the investigation of surrogate model-based bioinspired optimization algorithms, where different surrogate models might be better for particular problems.

In future work, the proposal’s implementation into a real-world OMR experimental platform is considered. Another future research direction is analyzing other surrogate model methods based on machine learning and deep learning techniques. Also, handling multiple control performance indexes based on multiobjective bioinspired optimization algorithms is considered.

## Figures and Tables

**Table 2:** Parameters of OMR.

Parameter	Description	Value
*r*	Wheel radius	0.0625 (m)
*L*	Wheel distance to the mass center	0.2870 (m)
*m*	OMR’s mass	16.319 (kg)
*J*	Wheel inertia	5.82 × 10^−4^ (${\mathrm{kg\cdot m}}^{2}$)
$I_{z}$	OMR’s inertia	0.5160 (${\mathrm{kg\cdot m}}^{2}$)

**Table 3:** Parametric functions of path-tracking problems.

Path	Name	Description
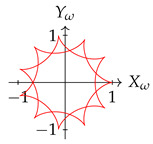	Hypocycloid	$t_{f}=120$ (s), $\tilde{R}=1$ (m), $\tilde{r}=\frac{\tilde{R}}{5.5}$ (m), $\tilde{A}=\tilde{R}-\tilde{r}$, $\tilde{B}=\frac{\tilde{R}-\tilde{r}}{\tilde{r}}$, $\tilde{p}=\frac{2\pi }{t_{f}}$ $x_{d}=\tilde{A}\cos\left(2\tilde{p}t\right)+\tilde{r}\cos\left(2\tilde{B}\tilde{p}t\right)$ $y_{d}=\tilde{A}\sin\left(2\tilde{p}t\right)-\tilde{r}\sin\left(2\tilde{B}\tilde{p}t\right)$ $\phi_{d}=0.4363\cos\left(2\tilde{p}t\right)$
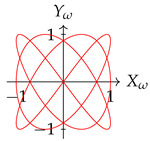	Lissajous	$t_{f}=60$ (s), ${\tilde{R}}_{1}={\tilde{R}}_{2}=1$ (m), $\tilde{a}=3$, $\tilde{b}=4$, $\tilde{p}=\frac{8\pi }{t_{f}}$ $x_{d}={\tilde{R}}_{1}\sin\left(\frac{\tilde{a}}{\tilde{b}}\tilde{p}t\right)$ $y_{d}={\tilde{R}}_{2}\tilde{p}\sin\left(\tilde{p}t\right)$ $\phi_{d}=0$

**Table 4:** Statistical results regarding $J_{T}$ for both experiments.

Experiment		Descriptive Statistics				Friedman Test	
	Approach	Best	Worst	Mean	std	Mean Ranks	*p*-Value
1	N-RTACG	2.1307 × 10^−7^	2.1307 × 10^−7^	2.1307 × 10^−7^	**0.0000 × $10^{0}$**	3	2.1906 × 10^−19^
	RTACG	2.1385 × 10^−7^	2.1385 × 10^−7^	2.1385 × 10^−7^	**0.0000 × $10^{0}$**	4	
	IACTA-GDM	**1.0418 × 10^−7^**	**1.0463 × 10^−7^**	**1.0441 × 10^−7^**	1.0653 × 10^−10^	1	
	SIACTA-RSM	1.7271 × 10^−7^	1.7273 × 10^−7^	1.7271 × 10^−7^	3.0259 × 10^−12^	2	
2	N-RTACG	9.3968 × 10^−2^	9.4085 × 10^−2^	9.4025 × 10^−2^	2.5022 × 10^−5^	4	7.1119 × 10^−18^
	RTACG	7.0024 × 10^−2^	7.0061 × 10^−2^	7.0040 × 10^−2^	**9.4029 × 10^−6^**	3	
	IACTA-GDM	4.9349 × 10^−3^	5.1654 × 10^−3^	4.9765 × 10^−3^	4.0126 × 10^−5^	1.7333	
	SIACTA-RSM	**4.7028 × 10^−3^**	**5.0632 × 10^−3^**	**4.8981 × 10^−3^**	9.2423 × 10^−5^	1.2667	

**Table 5:** Statistical results regarding ISU for both experiments.

Experiment			Descriptive Statistics			Friedman Test	
	Approach	Best	Worst	Mean	std	Mean Ranks	*p*-Value
1	N-RATCG	**1.5795 × 10^−1^**	**1.5795 × 10^−1^**	**1.5795 × 10^−1^**	**0.0000 × $10^{0}$**	1	2.1906 × 10^−19^
	RATCG	1.5800 × 10^−1^	1.5800 × 10^−1^	1.5800 × 10^−1^	**0.0000 × $10^{0}$**	3	
	IACTA-GDM	1.6682 × 10^−1^	1.6723 × 10^−1^	1.6703 × 10^−1^	9.5049 × 10^−5^	4	
	SIACTA-RSM	1.5797 × 10^−1^	1.5797 × 10^−1^	1.5797 × 10^−1^	5.0111 × 10^−10^	2	
2	N-RATCG	2.3297 × $10^{4}$	2.3768 × $10^{4}$	2.3490 × $10^{4}$	1.1141 × $10^{2}$	2	2.1906 × 10^−19^
	RATCG	**7.4827 × $10^{2}$**	**7.5726 × $10^{2}$**	**7.5208 × $10^{2}$**	**2.2351 × $10^{0}$**	1	
	IACTA-GDM	9.9324 × $10^{4}$	3.2594 × $10^{5}$	1.1573 × $10^{5}$	4.2063 × $10^{4}$	4	
	SIACTA-RSM	8.4261 × $10^{4}$	9.5455 × $10^{4}$	9.0526 × $10^{4}$	2.6083 × $10^{3}$	3	

**Table 6:** Statistical results regarding $S_{T}$.

	Descriptive Statistics				Wilcoxon Test	
Approach	Best	Worst	Mean	std	Symbol	*p*-Value
IACTA-GDM	3.7102 × 10^−2^	3.9189 × 10^−2^	3.7699 × 10^−2^	5.5312 × 10^−4^	−	1.7343 × 10^−6^
SIACTA-RSM	**1.3823 × 10^−2^**	**1.4201 × 10^−2^**	**1.4004 × 10^−2^**	**1.1408 × 10^−4^**	+	

**Table 7:** Statistical results regarding $T_{O}$ for both experiments.

Experiment		Descriptive Statistics				Wilcoxon Test	
	Approach	Best	Worst	Mean	std	Symbol	*p*-Value
1	IACTA-GDM	9.9976 × 10^−1^	**1.0000 × $10^{0}$**	9.9981 × 10^−1^	1.5974 × 10^−2^	−	1.7344 × 10^−6^
	SIACTA-RSM	**9.3936 × 10^−1^**	**1.0000 × $10^{0}$**	**9.6058 × 10^−1^**	**5.0659 × 10^−5^**	+	
2	IACTA-GDM	1.0564 × $10^{0}$	1.3642 × $10^{0}$	1.1139 × $10^{0}$	**5.5464 × 10^−2^**	−	1.7344 × 10^−6^
	SIACTA-RSM	**1.3187 × 10^−2^**	**7.7912 × 10^−1^**	**4.2277 × 10^−1^**	1.9871 × 10^−1^	+	

**Table 8:** Parametric functions for additional path.

Path	Name	Description
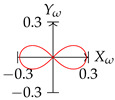	Lemniscate	$t_{f}=10$ (s), $\tilde{R}=0.2$ (m), $\tilde{p}=\frac{2\pi }{t_{f}}$ $x_{d}=\frac{\tilde{R}\sqrt{2}\cos\left(\tilde{p}t\right)}{1+\sin{\left(\tilde{p}t\right)}^{2}}$ $y_{d}=\frac{\tilde{R}\sqrt{2}\sin\left(\tilde{p}t\right)}{1+\sin{\left(\tilde{p}t\right)}^{2}}$ $\phi_{d}=\mathrm{atan}2\left(\frac{y}{x}\right)$

**Table 9:** Statistical results SIACTA-RSM vs. SIACTA-GPR.

Criterion		Descriptive Statistics				Wilcoxon Test	
	Approach	Best	Worst	Mean	std	Symbol	*p*-Value
$J_{T}$	SIACTA-RSM	**1.6719 × 10^−4^**	**1.9876 × 10^−4^**	**1.8118 × 10^−4^**	**7.6947 × 10^−6^**	+	1.7344 × 10^−6^
	SIACTA-GPR	2.0472 × 10^−4^	4.2912 × 10^−4^	2.2130 × 10^−4^	4.1726 × 10^−5^	−	
$S_{T}$	SIACTA-RSM	**1.4509 × 10^−2^**	**6.3955 × 10^−2^**	**2.6856 × 10^−2^**	**1.9132 × 10^−2^**	+	1.7344 × 10^−6^
	SIACTA-GPR	9.9327 × 10^−1^	1.1166 × $10^{0}$	1.0213 × $10^{0}$	2.4949 × 10^−2^	−	
$T_{O}$	SIACTA-RSM	**1.3013 × 10^−2^**	**1.2124 × 10^−1^**	**5.6136 × 10^−2^**	**2.7037 × 10^−2^**	+	1.7344 × 10^−6^
	SIACTA-GPR	1.4389 × 10^−1^	1.0001 × $10^{0}$	2.0708 × 10^−1^	1.5920 × 10^−1^	−	

## Data Availability

Data will be made available on request.
